# Multi-Modal Analysis of Programmed Cell Death Identifies Biomarkers and Informs Prognosis in Osteosarcoma

**DOI:** 10.3390/ijms27083431

**Published:** 2026-04-11

**Authors:** Xinyi Zou, Yuanfang Ru

**Affiliations:** College of Science, China Pharmaceutical University, Nanjing 211198, China; 13912608577@163.com

**Keywords:** osteosarcoma, programmed cell death, molecular subtypes, prognostic model

## Abstract

Osteosarcoma (OS), the most prevalent primary malignant bone tumor with a dismal prognosis, exhibits significant heterogeneity in programmed cell death (PCD) pathways, but its subtype-specific functional mechanisms remain poorly characterized. This study integrated PCD-related gene signatures to delineate molecular subtypes in OS via consensus clustering, successfully defining four distinct subtypes with divergent prognostic outcomes and immune microenvironments. Differential expression, functional enrichment, and protein–protein interaction (PPI) network analyses revealed subtype-specific PCD pathway associations (e.g., lysosome-dependent cell death, apoptosis, pyroptosis and anoikis), while comparative immune profiling and clinical characterization further refined subgroup identities. A robust prognostic risk model incorporating five pivotal genes (*SERPINE2*, *CBS*, *SQLE*, *UBE2D4*, and *S100A13*) and metastasis status demonstrated superior predictive performance in both training and external validation cohorts. These findings not only elucidate the functional architecture of PCD across OS molecular subtypes but also establish a clinically actionable model for precision risk stratification and tailored therapeutic strategies.

## 1. Introduction

Osteosarcoma (OS), the most frequent primary malignant bone tumor in adolescents, ranks as the eighth most common malignancy in this demographic [1]. Annually, China reports approximately 6000 new OS cases, underscoring its significant public health burden. Characterized by aggressive biological behavior and a high propensity for early metastasis [2], OS remains associated with suboptimal survival outcomes despite advances in multimodal therapy including surgical resection and adjuvant chemotherapy. Consequently, the 5-year overall survival rate persists below 70%, reflecting limited therapeutic efficacy. While emerging immunotherapeutic and targeted strategies present promising avenues for OS treatment [3], the absence of validated immune checkpoint inhibitors capable of effectively modulating the immunosuppressive tumor microenvironment remains a critical barrier. This therapeutic gap, coupled with the tumor’s inherent molecular heterogeneity, necessitates urgent identification of distinct OS subtypes, discovery of novel therapeutic targets, and development of robust prognostic models to enable precision oncology approaches for personalized treatment planning.

Programmed cell death (PCD) represents a genetically regulated process essential for tissue homeostasis in multicellular organisms [4]. Diverse PCD modalities, including apoptosis, necroptosis, ferroptosis, pyroptosis, parthanatos, autophagy, lysosome-dependent cell death, anoikis, aging-related cell death and NETosis, exhibit critical roles in tumorigenesis [5,6]. Increasing evidence suggests that PCD pathways significantly influence the malignant phenotype and therapeutic response of OS. Apoptosis proceeds via the following two conserved pathways: the intrinsic pathway, initiated by mitochondrial outer membrane permeabilization and cytochrome c release mediated by Bcl-2 family proteins, and the extrinsic pathway, triggered by death receptor (e.g., TNF-R1) engagement and formation of the death-inducing signaling complex (DISC) [7]. In OS, the frequent dysfunction of P53—a master regulator of this intrinsic pathway—disrupts mitochondrial-mediated apoptosis, creating a permissive environment for both tumor progression and the activation of alternative programmed cell death modalities. Necroptosis in OS is characterized by RIPK1-dependent inhibition of caspase-8 activation, driving inflammatory tumor microenvironments [8]. Ferroptosis is predominantly regulated by mitophagy and ferritinophagy in OS [9]. Pyroptosis, induced by the cleavage of the gasdermin (GSDM) superfamily, accelerates inflammatory cell death in OS via endoplasmic reticulum stress [10]. Parthanatos, driven by PARP-1 hyperactivation, correlates with OS proliferation, invasion, and metastasis [11]. Autophagy sustains OS progression by enhancing tumor survival, metastasis, and therapy resistance through PI3K/AKT/mTORC1 and ERK/MAPK signaling, positioning it as a promising therapeutic target [12]. Lysosome-dependent cell death involves lysosomal membrane permeabilization and hydrolytic enzyme leakage, causing cellular demise [13]. Anoikis, a detachment-induced PCD, facilitates OS invasion by disrupting cell–cell and cell–extracellular matrix adhesion [14]. Aging-related cell death is associated with altered intercellular communication and increased pro-inflammatory markers, potentially elevating cancer risk [15]. NETosis manifests as antitumor (N1) or protumor (N2) neutrophil subsets: N1 neutrophils enhance CD8^+^ T-cell-mediated antitumor immunity, whereas N2 neutrophils secrete chemokines (e.g., CCL2, CXCL1, and CXCL8) to promote angiogenesis and metastasis in the OS microenvironment [16]. Increasing evidence supported that these distinct modalities are increasingly recognized as critical determinants of therapeutic response and clinical outcome in OS. For instance, the susceptibility of tumor cells to lysosome-dependent cell death influences the efficacy of certain chemotherapeutic agents, while the modulation of anoikis resistance directly correlates with metastatic potential and poor prognosis. Similarly, the balance between N1 and N2 NETosis within the tumor microenvironment can either enhance therapeutic efficacy or contribute to immune evasion during immunotherapy. Despite growing recognition of their clinical significance, current research predominantly examines single PCD pathway (e.g., apoptosis or ferroptosis) in isolation and the association with the immunosuppressive tumor microenvironment in OS [17,18,19]. While several studies have broadly integrated PCD-related genes to identify prognostic biomarkers [20,21,22], subtype-specific mechanistic links between distinct PCD pathways and OS molecular subtypes remain unexplored.

To address this gap, we systematically curated signature genes from 10 PCD modalities and employed consensus clustering to define four OS molecular subtypes. Through Gene Set Enrichment Analysis (GSEA) and protein–protein interaction (PPI) network analysis, we elucidated the functional associations between specific PCD pathways and OS molecular subtypes. Subsequent Lasso-Cox regression model identified critical hub features, enabling the construction of a clinically robust prognostic model, which provides a mechanistic foundation for precision therapeutic strategies in OS.

## 2. Results

### 2.1. Identification of Four PCD-Specific Subtypes in OS

**Figure 1:**
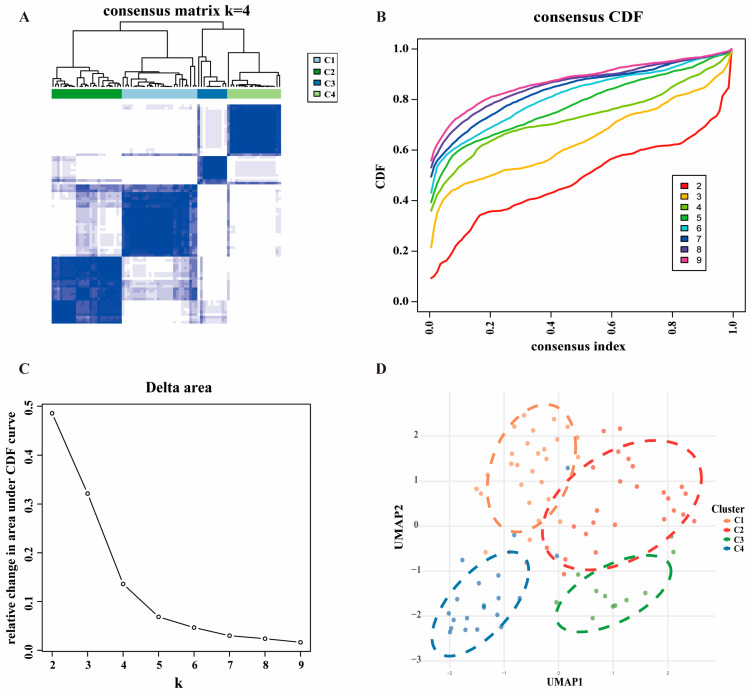
Landscape of the molecular cluster in the training set. (**A**) Consensus matrix at *k* = 4, sorted by the clustering results. (**B**) Cumulative distribution function (CDF) curves for different numbers of clusters. (**C**) Delta area plot. (**D**) Uniform Manifold Approximation and Projection (UMAP) plot for the four subtypes.

Supplementary Table S1 presents the comprehensive gene sets of the following 10 PCD modalities: autophagy (178 genes), apoptosis (193 genes), pyroptosis (92 genes), lysosome-dependent cell death (108 genes), ferroptosis (54 genes), NETosis (66 genes), parthanatos (64 genes), aging-related cell death (349 genes), anoikis (53 genes), and necroptosis (93 genes). After integration, a total of 1006 unique genes were derived. Consensus clustering analysis applied to the expression profiles of the training set (see Materials and Methods) identified four stable molecular subtypes, labeled C1, C2, C3, and C4 (Figure 1A). The optimal cluster number (*k* = 4) was determined using the cumulative distribution function (CDF) curve (Figure 1B) and the relative change in area (Figure 1C). Uniform Manifold Approximation and Projection (UMAP) dimensionality reduction confirmed distinct spatial segregation of the four subtypes (Figure 1D).

**Figure 2:**
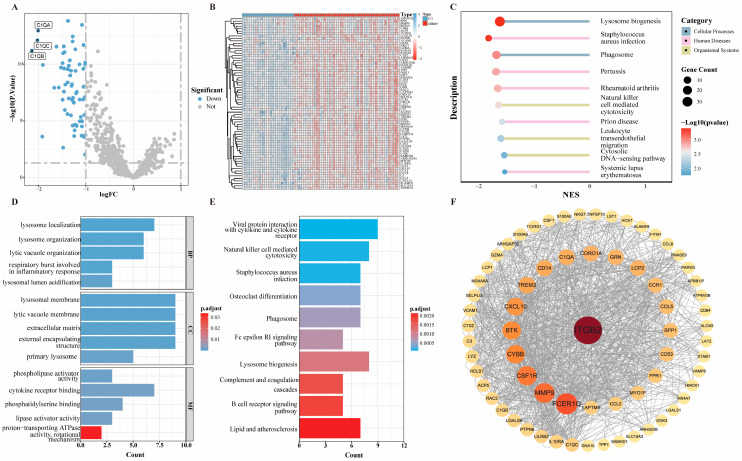
Identification of differentially expressed genes (DEGs), functional enrichment analysis and protein–protein interaction (PPI) network for subtype C1. (**A**) Volcano plot for differential expression analysis. (The horizontal dashed line represents *p* = 0.05, and the two vertical dashed lines represent the log_2_(fold change) thresholds of ±1.) (**B**) Heatmap of expression patterns for differentially expressed genes (DEGs) in the training set. (**C**) Pathways significantly enriched by Gene Set Enrichment Analysis (GSEA). (**D**) Bar graph of Gene Ontology enrichment analysis. (**E**) Bar graph of KEGG pathway distribution. (**F**) The PPI network of DEGs.

Differential expression analysis identified 70, 36, 139, and 50 subtype-specific DEGs for subtypes C1–C4, separately (Figure 2A and Figures S1A–S3A). Heatmap (Figure 2B)-visualizing expression patterns are presented for C1, while those for C2, C3, and C4 are shown in Figures S1B–S3B.

Subtype C1 featured 10 downregulated lysosome-associated genes, including *C1QA*, *C1QB*, *C1QC*, and *LAPTM5* [23], with GSEA exhibiting significant enrichment of the lysosome pathway (FDR < 0.05; Figure 2C). GO analysis also revealed distinct enrichment of lysosome-related terms across biological process, cellular component, and molecular function categories (e.g., lysosome localization, lysosome organization and lysosomal membrane) (Figure 2D), consistent with lysosomal membrane disruption as a core mechanism [24]. Furthermore, lysosome biogenesis and Phagosome (Figure 2E), displayed in the KEGG analysis, are closely associated with lysosomal membrane permeabilization. In addition, Yueshu Wu et al. [25] integrated single-cell and bulk RNA sequencing data, illustrating the activation of “lysosome” in OS samples.

Subtype C2 showed high expression of pyroptosis-related core genes, such as *AIM2* and *EGFR*. Relevant studies suggested that the pathways of pyroptosis mediated by pore-forming proteins are involved in the progress of OS [26,27,28]. Significant enrichment of the inflammatory pathway and responses to molecule of bacterial origin and lipopolysaccharide were observed, which trigger pyroptosis signaling and subsequently promote caspase-1 activation [29] (Figure S2D,E).

Subtype C3 displayed convergence of GSEA (Figure S3C) and KEGG (Figure S3E) on infection-related pathways (Tuberculosis, TNF signaling, and Efferocytosis), with TNF-α signaling (Figure S2D) identified as an apoptosis trigger [30]. GO analysis directly highlighted diverse regulations of the apoptotic signaling pathway (e.g., extrinsic and leukocyte-related), revealing the role of subtype C3 in apoptotic execution. In the context of OS, TP53 dysfunction can impair apoptotic pathway’s balance and subsequently contribute to the disease progression [31,32].

Subtype C4 contained significant enrichment of the extracellular matrix (ECM)–receptor interaction, focal adhesion and integrin signaling (Figure S4E). Meanwhile, the GO analysis (Figure S4C) revealed active remodeling of the extracellular matrix, linking this subtype to anoikis-associated processes [33,34]. Growing evidence [35,36,37] supported that the ECM cultivates tumorigenesis and metastasis in malignancies, including OS.

PPI network analysis identified hub proteins via betweenness centrality for C1–C4:

- C1: *ITGB2*, *FCER1G*, *MMP9*, *CSF1R*, *CYBB* (Figure 2F);
- C2: *COL3A1*, *CCL2*, *EGFR*, *MMP9*, *SPP1* (Figure S2F);
- C3: *MMP9*, *CXCL10*, *ITGB2*, *PTPRC*, *CTSB* (Figure S3F);
- C4: *EGFR*, *COL3A1*, *HAS2*, *MMP9*, *SPP1* (Figure S4F).

### 2.2. Clinical Correlation with the Different PCD Clusters

**Figure 3:**
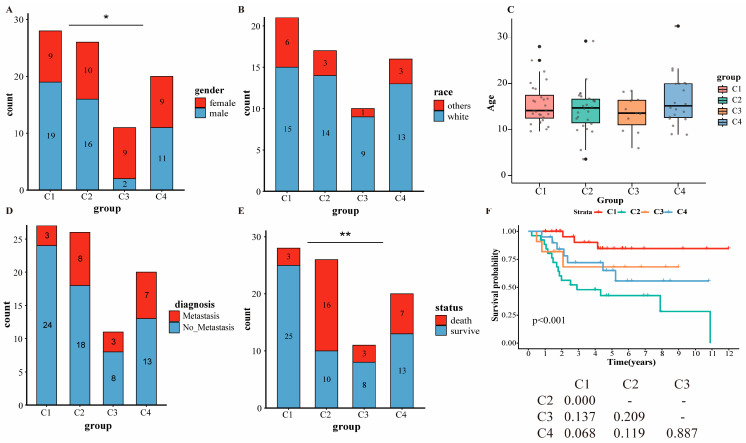
Clinical characteristics comparisons across osteosarcoma (OS) subtypes for training set. (**A**) Bar plot for sex. (**B**) Bar plot for race. (**C**) Boxplot for age. (The black dots represent outliers in the boxplot, and the gray dots represent the individual data points (raw age values) for each sample.) (**D**) Bar plot for tumor metastasis. (**E**) Bar plot for survival status. (**F**) Kaplan–Meier survival analysis. (Asterisks indicate statistical significance: * *p* < 0.05, ** *p* < 0.01.)

Clinical characteristics across molecular subtypes were analyzed. Figure 3A shows a significant difference in sex, with C3 exhibiting higher female predominance (81.8% vs. 37.8%, *p* < 0.05). No significant differences were observed in race (*p* = 0.650, Figure 3B), age (*p* = 0.538, Figure 3C), or tumor metastasis (*p* = 0.227, Figure 3D) among subtypes.

Overall survival probability was significantly higher in C1 versus all other subtypes (*p* = 0.001, Figure 3E). Kaplan–Meier analysis confirmed significant survival divergence across subtypes (*p* < 0.001, Figure 3F), with C2 demonstrating the poorest prognosis (median survival 2.907 years) and C1 showing the most favorable outcome (median survival not reached). C1 exhibited significantly improved survival versus C2 (*p* < 0.001), while C3 (*p* = 0.137) and C4 (*p* = 0.068) showed no significant survival difference compared with other subtypes.

### 2.3. Analysis of Immune Status in Different OS Subtypes

**Figure 4:**
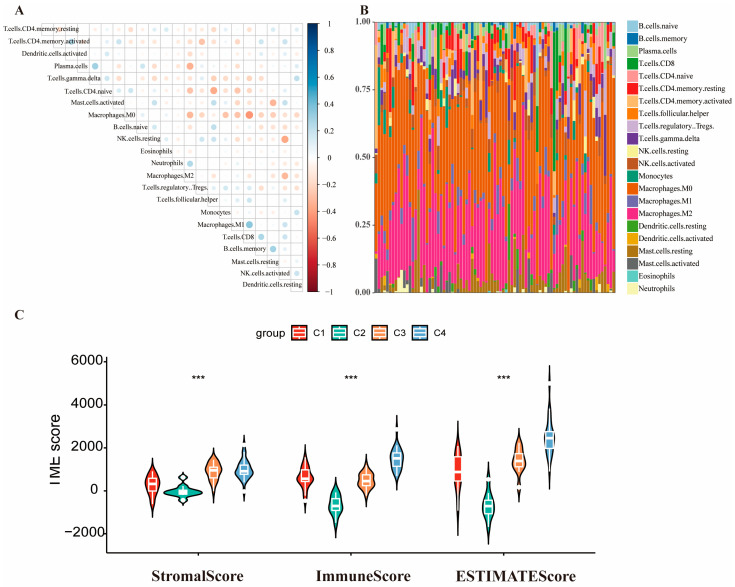
Immune landscape of OS molecular subtypes. (**A**) Correlation analysis of the immune cells. (Dot size represents the absolute value of the Pearson correlation coefficient.) (**B**) Proportions of immune cells in each sample. (**C**) Violin plot displaying the ESTIMATE score across the subgroups. (Asterisks indicate statistical significance: *** *p* < 0.001.)

Immune cell infiltration profiling across OS subtypes (22 cell types) was performed using “*CIBERSORT*” package (v0.1.0). Pearson correlation analysis revealed significant negative associations between M0 macrophages and M1 macrophages (ρ = −0.271, *p* = 0.012), M2 macrophages (ρ = −0.320, *p* = 0.003), CD8^+^ T cells (ρ = −0.421, *p* < 0.001), and monocytes (ρ = −0.272, *p* = 0.012; Figure 4A). Macrophages M0 and M2 constituted the dominant immune cell populations in each sample (Figure 4B). C1 exhibited significantly higher ESTIMATE scores (stromal: *p* < 0.001; immune: *p* < 0.001; and combined: *p* < 0.001) versus other subtypes (Figure 4C), correlating with improved overall survival. Pairwise comparisons revealed that, for StromalScore, subtypes C3 and C4 were significantly higher than C1 (*p* < 0.001) and C2 (*p* < 0.001). In contrast, for ImmuneScore and ESTIMATEScore, the only non-significant comparison was between C1 and C3 (ImmuneScore: *p* = 1.000; ESTIMATEScore: *p* = 0.180).

### 2.4. Construction and Validation of the Risk Score Model

**Figure 5:**
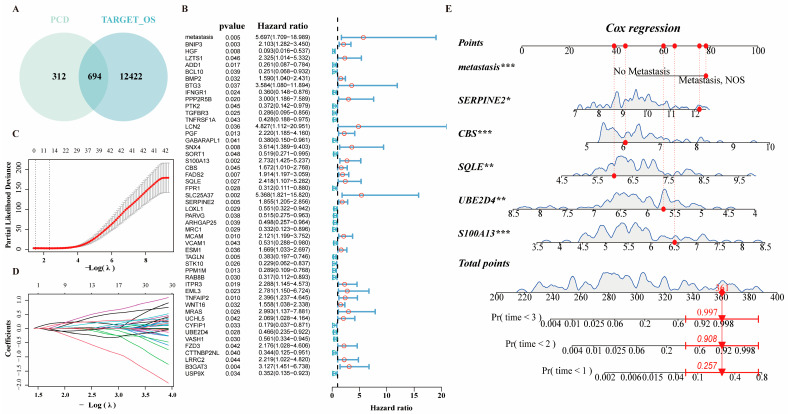
Construction of the prognostic model. (**A**) Venn diagram reveals 694 PCD-related genes in the training set. (**B**) Forest plot of the top 50 factors identified by univariate analysis. (**C**) Cross-validation curve for tuning the LASSO regression penalty parameter. (**D**) LASSO coefficient path plot. (**E**) A nomogram capable of predicting the prognosis of patients with OS. (Asterisks indicate statistical significance: * *p* < 0.05, ** *p* < 0.01, *** *p* < 0.001.)

Venn diagram analysis (Figure 5A) identified 694 available PCD-specific genes in the training set. Univariate Cox regression was subsequently performed on these 694 genes combined with five clinical factors (age, gender, race, metastasis, and tumor location), yielding 52 prognostic features (51 genes and one clinical variable: metastasis status) (Figure 5B). LASSO Cox regression (10-fold cross-validation) reduced these to 11 core genes and metastasis status (Figure 5C,D).

**Figure 6:**
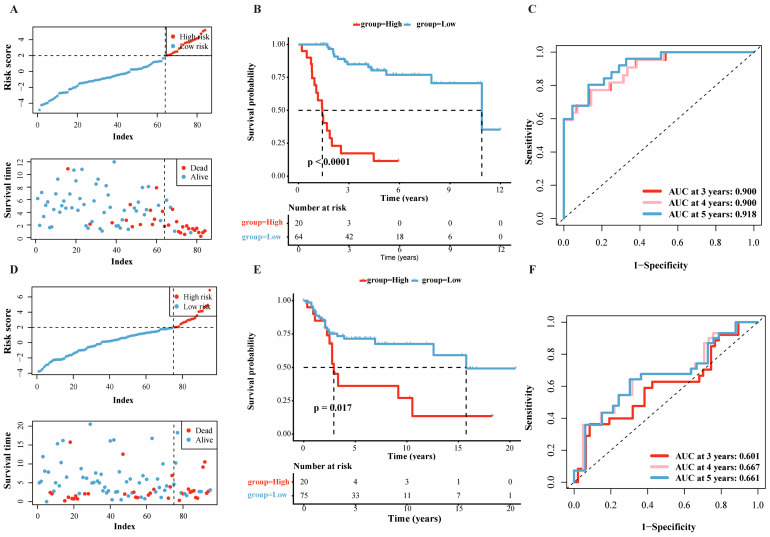
Training and external validation of the prognostic model. (**A**) Distribution of risk score between high- and low-risk groups in the training set. (In the upper panel, the horizontal dashed line represents the maximum risk score of the low-risk group (cutoff threshold), and the vertical dashed line represents the number of samples in the low-risk group (group boundary). In the lower panel, the vertical dashed line represents the number of low-risk samples, separating the survival time distributions of the low-risk and high-risk groups.) (**B**) Kaplan–Meier analysis of high- and low-risk groups in the training set. (**C**) The time-dependent ROC curve of nomogram in the training set. (**D**) Distribution of risk score between high- and low-risk groups in the external validation set. (Same dashed line explanations as in (**A**)). (**E**) Kaplan–Meier analysis of high- and low-risk groups in the external validation set. (**F**) The time-dependent ROC curve of nomogram in the external validation set.

Multivariate Cox regression incorporating these genes and the clinical variable yielded a final prognostic signature with significant predictors (*p* < 0.05): metastasis status, *SERPINE2*, *CBS*, *SQLE*, *UBE2D4*, and *S100A13* (Figure 5E). The risk score for each patient was derived using the prediction function within the “*glmnet*” package (v4.1-10). Patients in the training set were divided into high- and low-risk groups based on the optimal cutoff value of the risk score (Figure 6A, see Section 4). Kaplan–Meier curve demonstrated a significantly inferior prognosis for the high-risk group compared to the low-risk group (*p* <0.001, Figure 6B). The time-dependent ROC (Figure 6C) indicated the robustness of the prognostic model with AUC > 0.9 for 3, 4, and 5 years in the training data. External validation using combined GSE21257 and GSE39058 cohorts (see Section 4) confirmed significantly worse survival in high-risk patients (*p* = 0.017, Figure 6D,E). The ROC curve values (Figure 6F) of 3, 4 and 5 years were 0.601, 0.667 and 0.661, respectively, indicating the better prediction of model for long-term outcomes. Compared with the prognostic model reported by Zhang et al. [21], our prognostic model demonstrated superior predictive performance. In the training cohort (TARGET-OS), the 1-, 3-, and 5-year AUCs for this nomogram were 0.949, 0.900, and 0.918, respectively, compared with 0.88, 0.87, and 0.91 from Zhang et al. In the validation cohort (GSE21257), the corresponding AUCs for the proposed model were 0.663, 0.688, and 0.739, whereas the previous model exhibited a marked decline from 0.80 at 1 year to 0.56 and 0.62 at 3 and 5 years. Additionally, our prognostic model exhibited clearer stratification of high- and low-risk groups.

## 3. Discussion

In this study, 10 PCD modalities were comprehensively employed to identify four OS subtypes using consensus clustering method. Differential expression analysis, functional enrichment analysis and PPI network analysis were further performed to analyze the connection between PCD modalities and OS subtypes. Subsequently, immune profiles and clinical characteristics were compared across the subgroups. Furthermore, we screened crucial genes and incorporated relevant clinical characteristics to establish a prognostic risk scoring model. An external validation cohort was used to confirm the robust performance of the prognostic model.

Although previous integrative studies [21,22] have categorized OS into broad subgroups based on PCD patterns, they have not sufficiently elucidated the precise associations between specific PCD modalities and the distinct biological characteristics or clinical behavior of individual subgroup. In this study, we conducted consensus clustering analysis on expression profiles of 10 well-defined PCD modalities, to identify the optimal number of molecular subgroups. Furthermore, comprehensive functional enrichment analyses were performed to delineate the dominant PCD mechanisms underlying each subtype. C1 displayed features of lysosomal membrane disruption, leading to cell death [24], which was corroborated by concordant patterns in overall survival outcomes and tumor microenvironment composition. C2 exhibited significant enrichment of infection-related pathways, suggesting a strong link with pyroptosis, particularly via molecule of bacterial origin and lipopolysaccharide [29]. C3 was predominantly associated with enhanced apoptosis mediated by inflammatory cytokines release, which was consistent with the well-established role of these cytokines as potent inducers of apoptosis [38]. For C4, pronounced enrichment of the extracellular matrix (ECM)–receptor interaction transduces pro-survival signals (e.g., via FAK-PI3K-AKT pathway) that suppress anoikis [39]. This refined molecular stratification not only clarifies the intrinsic mechanisms links between specific PCD pathways and OS heterogeneity but also establishes a theoretical framework to guide the development of subtype-tailored therapeutic strategies.

In addition, comparisons of clinical information between OS subtypes showed that age, race and metastasis were not statistically different. However, sex showed a significant difference across subtypes, and the female patients were mainly distributed in C3. There were statistically more survival samples in C1 than in the other subtypes. As expected, Kaplan–Meier analysis demonstrated that C1 exhibited the more favorable outcome than C2.

The correlations among ESITMATE immune scores demonstrated that macrophage M0 is negatively associated with macrophages M1, M2, T cells CD8, and monocytes. Macrophages M0 and M2 accounted for dominant immune cell populations in each sample. Yu et al. analyzed single-cell RNA datasets and bulk RNA datasets investigating the same immune landscape of the TME in OS [40]. The ESTIMATE algorithm revealed that the stromal, immune, and combined scores were significantly elevated in C4, whereas they were markedly depressed in C2.

Through univariate Cox regression, LASSO-Cox regression and multivariate Cox regression, five hub genes (*SERPINE2*, *CBS*, *SQLE*, *UBE2D4*, and *S100A13*) were identified and used to construct the prognostic risk scoring model. *SERPINE2* (serpin family E member 2, also known as protease nexin-1) functions as a serine protease inhibitor [41]. In this study, *SERPINE2* expression in the high-risk cluster is statistically higher than the low-risk group, suggesting its potential role in enhancing local invasiveness and serving as a therapeutic target within the tumor microenvironment [42]. The oncogenic significance of *SERPINE2* in osteosarcoma is well-supported by previous studies. Mao and Wang reported that *SERPINE2* was upregulated in osteosarcoma, promoted cell proliferation and drug resistance, and correlated with poor prognosis [43]. Similarly, Chen et al. demonstrated that *SERPINE2* knockdown inhibited tumor progression and enhanced cisplatin sensitivity [44]. Existing studies have shown that the *CBS* gene exerts a dual role across different cancer types [45]. In colon or ovarian cancer, high *CBS* expression drives both angiogenesis and tumor proliferation, which is consistent with the function of *CBS* observed in our OS study. In our analysis, high *SQLE* expression was a strong independent risk factor in OS. This aligns with a recent multi-omics study by Wang et al., which integrated machine learning, single-cell RNA sequencing, and in vivo pharmacological experiments to demonstrate that *SQLE* is a metabolic vulnerability in high-risk osteosarcoma [46]. As reviewed by Mao et al., the ubiquitin–proteasome system plays a critical role in osteosarcoma development and represents a promising therapeutic target [47]. In this context, *UBE2D4* was found to be silenced in the osteosarcoma (OS) samples, also suggesting its potential function in prohibiting local invasiveness and serving as a candidate therapeutic target. *S100A13*, the member of S100 family, was evidenced as a risk factor on tumor progression and therapy resistance [48,49]. Li et al. and Tang et al., via multi-omics and experimental validation, revealed that *S100A13* maintains cancer stemness, promotes tumor progression and metastasis, and is associated with poor prognosis in osteosarcoma [50,51].

This study acknowledges several limitations. First, the analyses were derived from retrospective datasets sourced from public databases. Although rigorous bioinformatic methods and an external validation cohort were implemented, the inherent selection bias and incomplete clinicopathological annotation, such as variability in chemotherapy protocols, absence of longitudinal imaging data, and lack of treatment response metrics, may constrain the model’s external validity. Prospective, multi-center studies with standardized treatment regimens and comprehensive clinical documentation are essential to substantiate its real-world applicability. Second, immune infiltration analysis was performed using CIBERSORT on bulk RNA-seq data, an approach with known limitations and low resolution; therefore, our interpretations of immune cell differences should be viewed with appropriate caution, and future studies employing higher-resolution methods such as single-cell RNA sequencing (scRNA-seq) will be necessary to validate these findings. Third, while a robust prognostic gene signature was established, the functional roles of most signature genes in OS pathogenesis remain incompletely elucidated. Specifically, the dynamic crosstalk among different PCD pathways within the tumor microenvironment and their integrated influence on cellular fate necessitate further mechanistic exploration through well-designed in vitro assays and models. Fourth, despite promising performance in computational validation, clinical translation of the model faces non-trivial barriers. These include the requirement for prospective validation in diverse patient cohorts, standardization of assay methodologies (e.g., RNA-seq protocols), and rigorous assessment of clinical feasibility, cost-effectiveness, and ethical considerations. Addressing these challenges through well-designed translational studies will be pivotal for advancing precision oncology in osteosarcoma.

## 4. Materials and Methods

### 4.1. Data Acquiring and Processing

The transcriptome expression data from TARGET-OS, GSE21257, and were downloaded from the UCSC Genome Browser “http://genome.ucsc.edu (accessed on 5 January 2026)” and NCBI GEO “https://www.ncbi.nlm.nih.gov/ (accessed on 5 January 2026)” databases. The TARGET-OS, serving as the training set, comprised 88 OS patients. After excluding three cases with missing survival status, 85 samples were retained for the analysis. The GSE21257 and GSE39058 datasets were used for validation, containing 53 and 42 samples respectively. During the data preprocessing phase, lowly expressed genes were filtered, and the “*limma*” package (v3.62.2) was employed to correct potential batch effects among the different datasets. A total of 1006 PCD -related genes were acquired from the GSEA database “www.gsea-msigdb.org/gsea/ (accessed on 7 January 2026)” and the relevant literature [22]. Detailed information on the genes was presented in the Supplementary Materials.

### 4.2. Consensus Clustering Analysis

Consensus clustering was applied to define molecular subtypes of OS using the expression data from the training set. Optimal cluster number (*k*) was determined using the “*ConsensusClusterPlus*” R package (v1.62.0) through iterative resampling (100 iterations, 80% subsampling) and the “Consensus metric”. Hierarchical clustering employed complete linkage with Pearson correlation as the distance metric. Subtype stability was validated via consensus matrix visualization and silhouette width analysis. Molecular subtype assignments were projected onto a two-dimensional UMAP embedding for dimensionality reduction and visualization.

### 4.3. Identification of Differentially Expressed Genes

Differentially expression analysis was performed using the “*limma*” package (v3.62.2) to identify subtype-specific genes across molecular subtypes in the TARGET-OS cohort. DEGs were defined as |log_2_(fold change)| > 1 and FDR < 0.05 after Benjamini–Hochberg correction. Differential expression signatures were visualized via volcano plots and hierarchical heatmaps generated with “*ggplot2*” (v4.0.0), with genes and samples clustered using Euclidean distance and complete linkage.

### 4.4. Functional Enrichment Analysis

Functional enrichment analysis was performed using GO and KEGG pathways to characterize subtype-specific biological functions and canonical signaling pathways enriched in the DEGs for each OS molecular subtype. Enrichment significance was assessed using FDR < 0.05, and the GO terms and KEGG pathways were visualized via the “*clusterProfiler*” package v4.6.2).

### 4.5. Establishment of PPI Network

PPI network construction was performed using the STRING database “ https://string-db.org/, (accessed on 28 January 2026)” with a minimum interaction confidence score of 0.4 (medium confidence). Subsequent network analysis was conducted via the “*CytoNCA plugin*” (v2.2) in “*Cytoscape*” (v3.10.3), where node betweenness centrality was calculated to identify hub proteins. Network visualization employed a radial layout with nodes arranged in concentric circles by descending centrality scores.

### 4.6. Analysis of Immune Microenvironment

The tumor microenvironment (TME) composition, defined by immune cell infiltration and stromal interactions, critically influences diagnostic accuracy, prognostic stratification, and therapeutic responsiveness in OS [52]. Immune cell infiltration profiles were quantified using the “*CIBERSORT*” algorithm (v0.1.0) for each OS sample. Stromal score, immune score, and tumor purity were computed for each sample using the “*ESTIMATE*” package (v1.0.13) based on gene expression profiles, enabling quantitative assessment of TME composition. Subtype-specific differences in immune score were visualized using violin plots, with statistical significance determined by Kruskal–Wallis tests (*p* < 0.05).

### 4.7. Construction of the Prognostic Risk Model

Univariate Cox regression analysis was applied on the training set to select the prognostic features with FDR < 0.05. LASSO Cox regression (10-fold cross-validation) implemented via the “*glmnet*” package (v4.1-10) reduced 51 candidate genes to 11 core genes. The optimal regularization parameter λ was determined by minimizing the mean cross-validated error (λ_min), which yielded the most parsimonious model with robust predictive performance. A final multivariate Cox regression model was constructed using the selected genes and clinical variables to evaluate the prognostic risk scores. Furthermore, patients were stratified into high- and low-risk groups using the optimal cutoff value of the risk score, which was determined by maximizing the log-rank test statistic in the training set. Kaplan–Meier survival analysis was employed to assess the predictive performance of the prognostic risk model. External validation was performed using the combined datasets of GSE21257 and GSE39058, totaling 95 OS samples, to evaluate model generalizability.

### 4.8. Statistics

Statistical analyses were conducted in R (version 4.1.3). Group comparisons were made as follows: the chi-square test for categorical variables and either ANOVA or the Kruskal–Wallis test for continuous variables, depending on whether parametric assumptions were met. Statistical significance was defined as *p* < 0.05. In graphical presentations, asterisks indicate significance levels: * *p* < 0.05, ** *p* < 0.01, and *** *p* < 0.001.

## Data Availability

The original contributions presented in this study are included in the article/Supplementary Material. Further inquiries can be directed to the corresponding author.

## Supplementary Materials

The following supporting information can be downloaded at https://www.mdpi.com/article/10.3390/ijms27083431/s1.

## Abbreviations

The following abbreviations are used in this manuscript:

CDF	Cumulative distribution function
DEGs	Differentially expressed genes
DISC	Death-inducing signaling complex
FDR	False discovery rate
GO	Gene ontology
GSDM	Gasdermin
GSEA	Gene set enrichment analysis
KEGG	Kyoto Encyclopedia of Genes and Genomes
N1	Antitumor
N2	Protumor
OS	Osteosarcoma
PCD	Programmed cell death
PPI	Protein–protein interaction
TME	Tumor microenvironment
TRIM	Tripartite motif
UMAP	Uniform manifold approximation and projection
