# Supplementary material for: Multi-Modal Analysis of Programmed Cell Death Identifies Biomarkers and Informs Prognosis in Osteosarcoma
Source: Int J Mol Sci. 2026 Apr 11;27(8):3431. doi: 10.3390/ijms27083431 (PMC13116443; doi:10.3390/ijms27083431)
Supplement: Supplementary file 1 [file ijms-27-03431-s001.zip › supplemetary captions.pdf]

Figure S1. Batch effect removal results for the TARGET-OS, GSE21257 and GSE39058 datasets. (**A**) Boxplot before batch effect removal. (**B**) Boxplot after batch effect removal. (**C**) PCA plot before batch effect removal. (**D**) PCA plot after batch effect removal.

Figure S2. Identification of DEGs, functional enrichment analysis and PPI network for subtype C2. (**A**) Volcano plot for differential expression analysis. (The horizontal dashed line represents p = 0.5, and the two vertical dashed lines represent the log2(fold change) thresholds of ±1.) (**B**) Heatmap of expression patterns for DEGs in the training set. (**C**) Pathways significantly enriched by GSEA. (**D**) Bar graph of Gene Ontology enrichment analysis. (**E**) Bar graph of KEGG pathway distribution. (**F**) The PPI network of DEGs.

Figure S3. Identification of DEGs, functional enrichment analysis and PPI network for subtype C3. (**A**) Volcano plot for differential expression analysis. (The horizontal dashed line represents p = 0.5, and the two vertical dashed lines represent the log2(fold change) thresholds of ±1.) (**B**) Heatmap of expression patterns for DEGs in the training set. (**C**) Pathways significantly enriched by GSEA. (**D**) Bar graph of Gene Ontology enrichment analysis. (**E**) Bar graph of KEGG pathway distribution. (**F**) The PPI network of DEGs.

Figure S4. Identification of DEGs, functional enrichment analysis and PPI network for subtype C4. (**A**) Volcano plot for differential expression analysis. (The horizontal dashed line represents p = 0.5, and the two vertical dashed lines represent the log2(fold change) thresholds of ±1.) (**B**) Heatmap of expression patterns for DEGs in the training set. (**C**) Pathways significantly enriched by GSEA. (**D**) Bar graph of Gene Ontology enrichment analysis. (**E**) Bar graph of KEGG pathway distribution. (**F**) The PPI network of DEGs.

Table S1. 10 types of programmed cell death patterns related gene sets.
