# Supplementary figures and images for: Multi-Modal Analysis of Programmed Cell Death Identifies Biomarkers and Informs Prognosis in Osteosarcoma
Source: Int J Mol Sci. 2026 Apr 11;27(8):3431. doi: 10.3390/ijms27083431 (PMC13116443; doi:10.3390/ijms27083431)

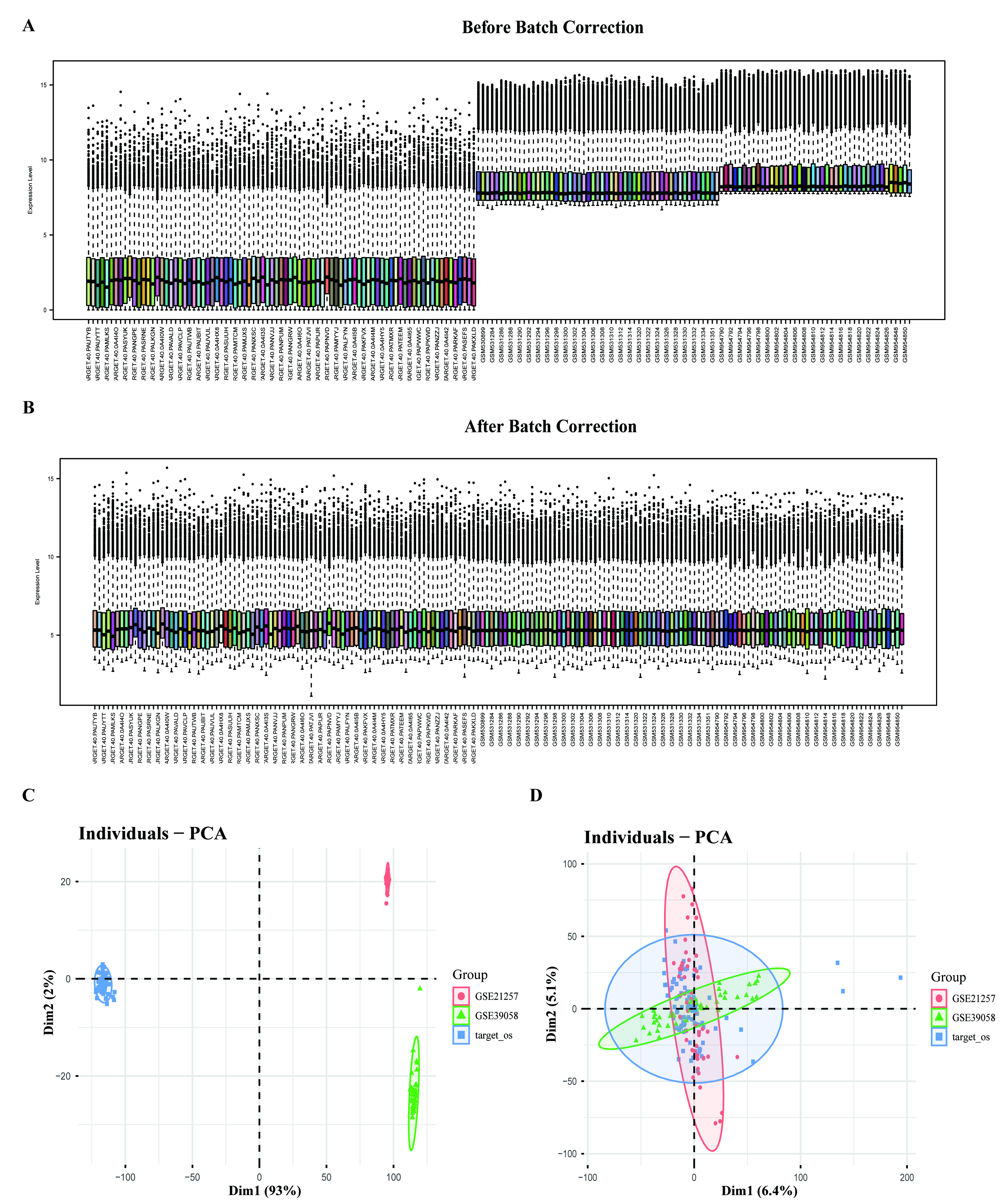

Supplement: Supplementary file 1 [file ijms-27-03431-s001.zip › FigS1.tif]

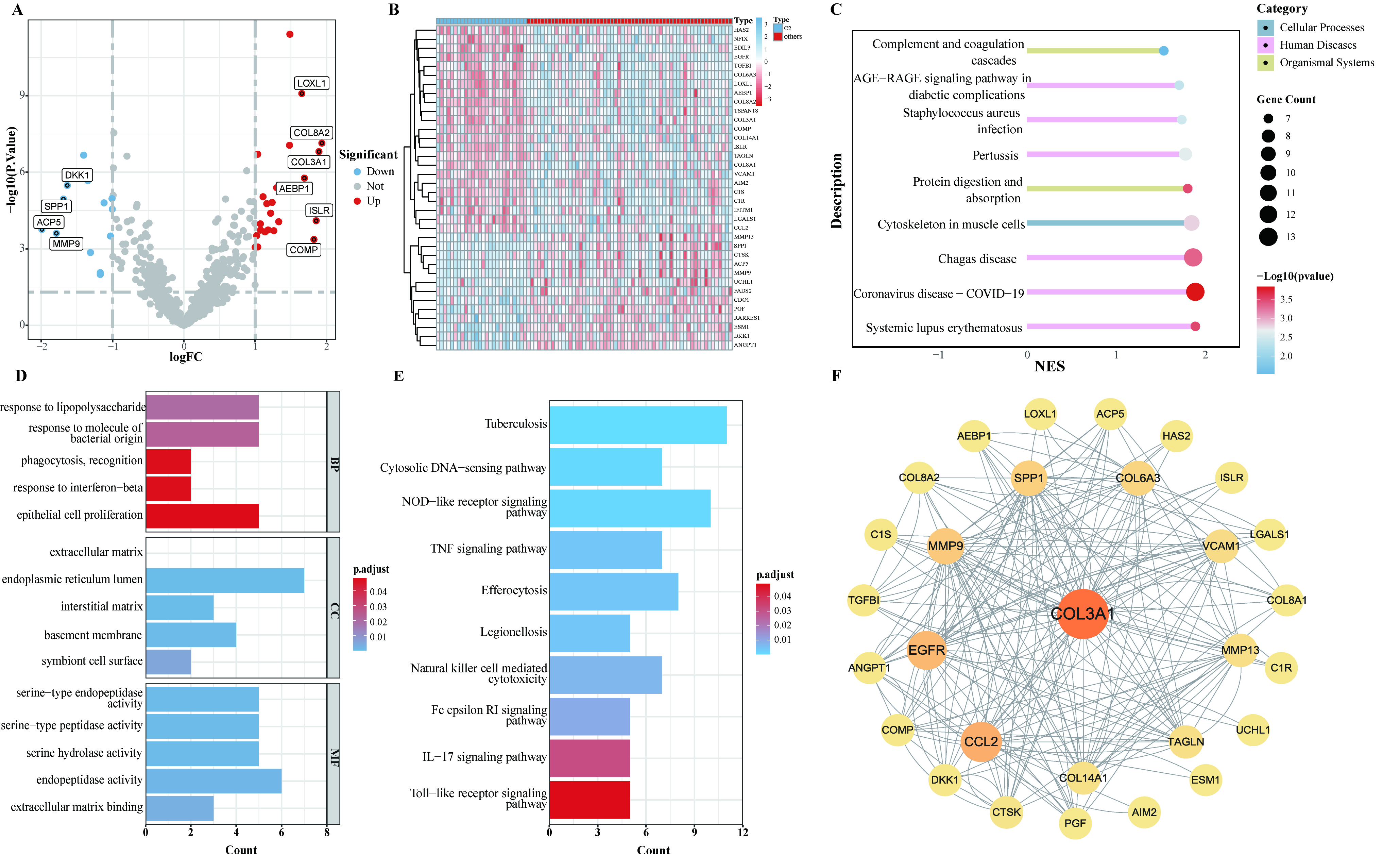

Supplement: Supplementary file 1 [file ijms-27-03431-s001.zip › FigS2.tif]

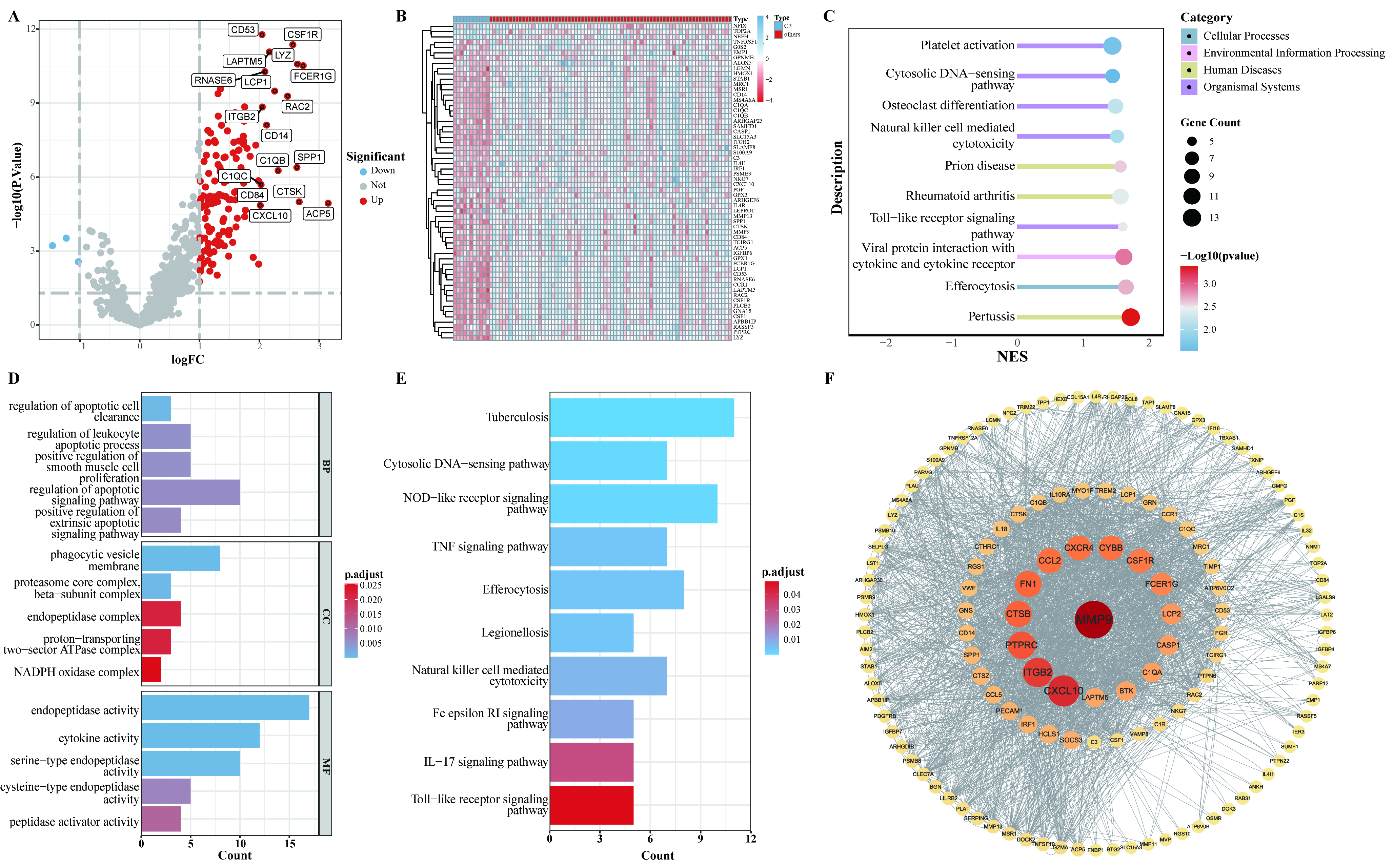

Supplement: Supplementary file 1 [file ijms-27-03431-s001.zip › FigS3.tif]

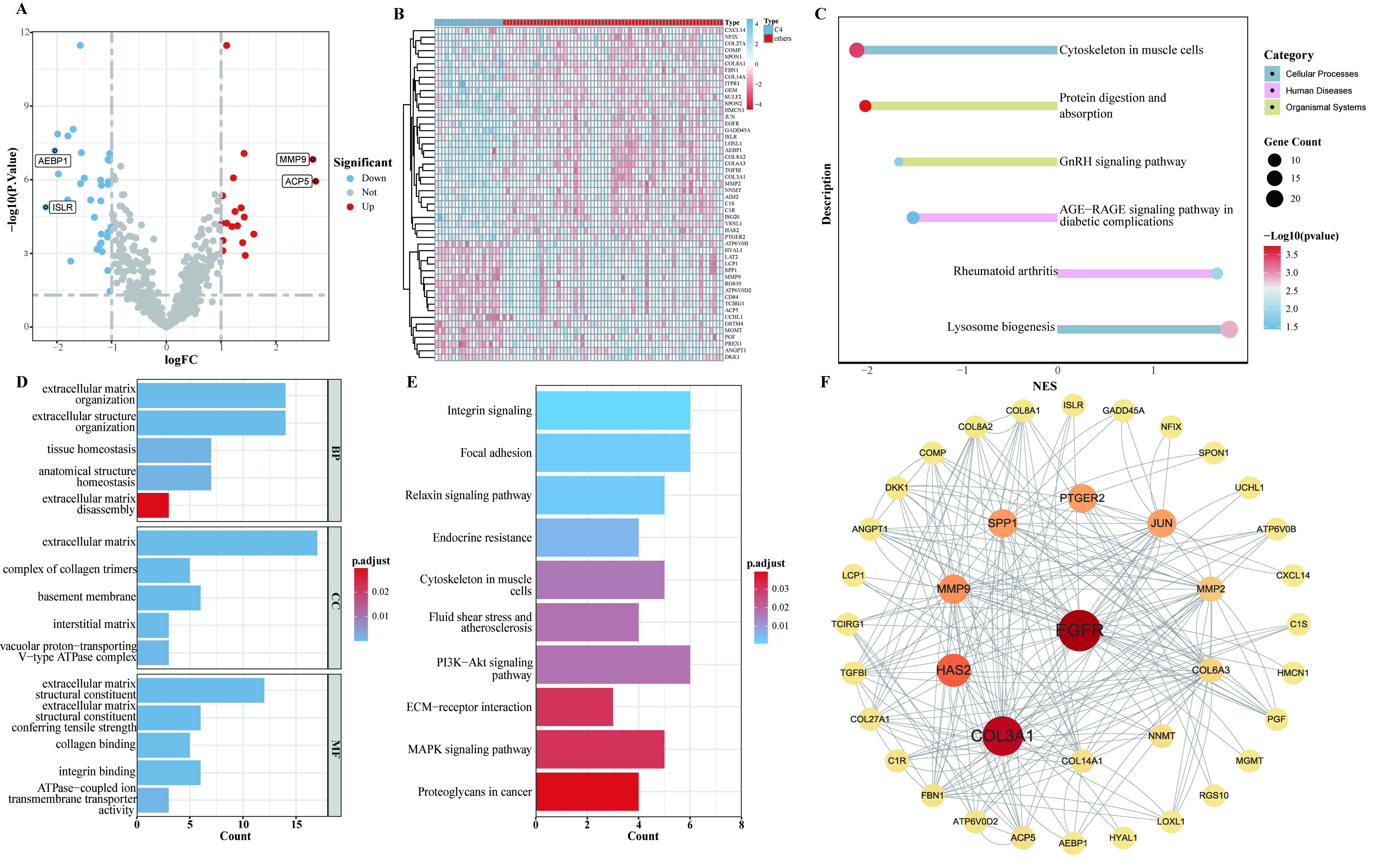

Supplement: Supplementary file 1 [file ijms-27-03431-s001.zip › FigS4.tif]
